# Associations between maturation assessment methods and growth-related injury occurrence in a German Bundesliga youth football academy

**DOI:** 10.1038/s41598-026-69051-y

**Published:** 2026-09-03

**Authors:** Felix Laukhardt, Tobias Engeroff, Sebastian Schneider, Christian Haser, Daniel Niederer, David Groneberg, Winfried Banzer

**Affiliations:** 1 Institute of Occupational, Social and Environmental Medicine, Goethe University, Theodor-Stern-Kai 7, Building 9B, 60590 Frankfurt, Germany; 2 Medical Department, Eintracht Frankfurt, Frankfurt, Germany

**Keywords:** Growth, Injury, PHV, Prevention, Football, Soccer, Diseases, Health care, Medical research, Risk factors

## Abstract

Peak height velocity (PHV) marks a sensitive period for adolescent athletes with increased injury risk. This prospective observational study explores how different maturation assessment methods are associated with injury occurrence, particularly growth-related injuries, during PHV in elite youth football players. Maturation of male U9-U16 players from the academy of a German Bundesliga club was assessed four times over one year using nine different methods (Mirwald, Moore, Fransen: ± 6/12 months, Khamis-Roche 85–96%/88–96%, ultrasonic BAUSport). Maturation was categorised into three periods relative to players’ PHV (Pre-PHV, PHV, Post-PHV). For each assessment-quarter, injury occurrence was categorised as no injury, growth-related, non-contact, or contact. Injury occurrence across maturation periods was analysed in a Bayesian multinomial regression using data from 205 players (619 assessments). In this single-academy cohort, only the ultrasonic BAUSport-defined PHV period was associated with higher odds of growth-related injury, as results reveal lower odds versus no injury in Pre- and Post-PHV periods (OR = 0.33 and 0.17) relative to PHV. Most methods demonstrated higher odds of non-contact injury versus no injury with progressive maturation, while contact injuries did not differ between maturation periods. This preliminary association warrants evaluation in larger longitudinal cohorts with exposure data before informing monitoring or prevention practice.

**Clinical trial registration:** The study was registered at the German Clinical Trial Register (www.drks.de; DRKS00036919).

## Introduction

Growth marks an essential characteristic in adolescence. Over the course of an adolescent’s physical development, the rate of growth gradually accelerates until puberty, when it reaches a peak (peak height velocity; PHV)^1^. The time of PHV represents a particularly sensitive period, as rapid changes in the longitudinal growth can drastically increase the risk of injury, particularly growth-related injury^2^, especially when they are exposed to high volumes of intensive sport, as it is the case in football academies. Several studies associate the PHV with a higher injury occurrence^3–5^, mainly located at the lower limbs^6^. However, the exact influence of PHV timing and maturation in general on players’ injuries is still not entirely clear^2,7^. One aspect that makes comparing the results of different studies difficult is the variation in methods used to estimate maturation and predict the timing of PHV^2^.

To evaluate maturation in children and adolescents, radiography is considered the gold-standard method. However, this method has several disadvantages (radiation exposure, cost, time intensity, and the requirement for a high level of expertise) that make repeated maturation estimations throughout the year impractical^2^. For these reasons, established methods in football practice are mostly equations incorporating anthropometric data^2^. Within those, there are equations calculating the actual percentage of the predicted adult height (%PAH) and equations calculating the age at PHV (APHV)^2^. The most common equation using the %PAH approach is the Khamis-Roche method^8^. This method estimates the maturation status by the proportion of the calculated adult height. Common methods using the APHV approach are the Mirwald method^9^, the Moore method^10^ and the Fransen method^11^. From the difference of the chronological age and the estimated APHV, a maturity offset is calculated, indicating a player’s maturation status. A more recent method for estimating maturation is the ultrasound-based assessment of the hand^12^, aiming to approximate radiographic gold-standard methods while being more feasible for the practical setting of football academies^2^. This method has been validated for skeletal age in Spanish male and female athletes aged nine to seventeen years^12^, and intra-rater and intra-device reliability have been demonstrated^12,13^. However, no study has yet evaluated the validity of its adult height prediction, from which the at-risk periods are derived. A natural limitation of such ultrasonic methods is that they measure bone maturation stages only indirectly through acoustic properties, not through direct visualisation.

The evaluation of the PHV timing is the primary purpose of equations using the APHV approach (e.g., Mirwald, Moore, Fransen). Methods using the %PAH approach (e.g., Khamis-Roche, ultrasonic method) define a specific range of achieved proportion of the PAH, in that the PHV is most likely to occur. Concluding this, some practically established approaches like the Khamis-Roche method and the ultrasonic method are more suitable for maturity estimation, whereas other approaches like the equations from Mirwald, Moore, and Fransen are more accurate in detecting the timing of PHV.

What all the established methods have in common is that they define a maturational range, referred to in this article as the Red-Flag range, which indicates the time when PHV is expected to occur and, by extension, the time when players are more likely to suffer a growth-related injury. Those ranges are practice- or literature-derived operational definitions, not proven biological boundaries. For methods using the APHV approach, the Red-Flag range is usually defined as either one year^14,15^ or six months^5,16^ around the calculated APHV. Methods using the %PAH approach, usually define a percentage range of the predicted adult height as Red-Flag range. The ultrasonic method uses a range between 85 and 96%. This range is specified by the manufacturer and not yet empirically justified. The Khamis-Roche method has several ranges found in the literature^4,15,17^. Often used ranges are between 88% and 96%^17^ and between 85 and 96%^15^, matching the ultrasonic method.

To prevent injuries related to growth, it is important to detect the PHV of adolescent athletes. Reliable detection of the PHV forms the basis for further targeted preventive measures. Given the many different methods used to estimate PHV, this exploratory study examines commonly used approaches in youth football academies with regard to their association with the occurrence of injuries, especially growth-related injuries, and describes how the Red-Flag ranges of these methods relate to growth-related injury occurrence.

## Materials and methods

### Study design and ethical aspects

We conducted a prospective observational study that was approved by the local Ethics Committee and registered at the German Clinical Trial Register. The measurements were carried out according to the ethical standards set by the Declaration of Helsinki (Version Fortaleza, 2013). Each player had a written informed consent signed by their parents or legal guardian before participating in the study.

### Participants

Participants were male football players of an elite youth academy from a German Bundesliga club in the age groups U9 to U16. All players participated in the regular team trainings and on average played one match per week, unless they were injured. Injured players were also included for measurements.

### Assessment of maturation and injuries

Maturation of the players was assessed four times over the period of one year. Assessments included mass, standing height and sitting height, as well as a scan of the left hand and wrist using the ultrasonic BAUSport (SonicBone Medical Ltd., Tel-Aviv, Israel; serial number: 000020018; software version 1.0.0.31) device and were conducted at a standardised time point before team training in the afternoon. In addition to the measurements, players’ dates of birth, the height of their biological parents (self-reported), and the dates of measurement were collected.

For the standing height measurements, players stood upright, barefoot and with their feet together, having their head in the Frankfort plane and took a deep breath. Standing height was measured to the nearest 0.1 cm as the distance from the surface to the vertex of the head using a stationary stadiometer (seca 216, PHG seca GmbH & Co. KG., Hamburg, Germany). For the sitting height measurements, players sat upright on a box with their head in the Frankfort plane and taking a deep breath. Sitting height was measured to the nearest 0.1 cm as the distance from the upper side of the box to the vertex of the head using the same stadiometer as for the standing height. Mass measurements were performed in football shorts and shirt, but without shoes, shin guards or other football equipment, measuring to the nearest 0.1 kg using a portable scale (seca 799, PHG seca GmbH & Co. KG., Hamburg, Germany). The ultrasonic scans on the BAUSport device were performed at three locations on the left hand, scanning the wrist, the third phalanx of the middle finger, and the metacarpal bones. Before measurements with the ultrasonic device, calibration was performed by placing two sticks of different length under each scanning location of the device. Measurements were performed by the same examiner, who had undergone specific training with a member of staff from SonicBone to ensure proper execution. Intra-rater reliability was not assessed in this study, but an earlier investigation reported good intra-rater reliability^13^. During the assessments, the propagation speed of high frequency waves from a short ultrasound pulse through bone as well as the attenuation of the pulse amplitude, were measured. Using those parameters, the biological age and predicted adult height were automatically calculated by the BAUSport software based on the Tanner-Whitehouse equation^18^. Measurements were carried out in accordance with the manufacturer’s instructions, and scans that failed were repeated. The operator was blinded to the players’ prior measurements.

After the measurements, players were categorised into their respective maturation period (Pre-PHV, PHV and Post-PHV) for each applied method. The used methods and their Red-Flag ranges were the Mirwald method (APHV ± 6 months (Mirwald short); APHV ± 12 months (Mirwald long)), the Moore method (APHV ± 6 months (Moore short); APHV ± 12 months (Moore long)), the Fransen method (APHV ± 6 months (Fransen short); APHV ± 12 months (Fransen long)), the Khamis-Roche method (88–96% PAH (Khamis-Roche short); 85–96% PAH (Khamis-Roche long)) and the ultrasonic BAUSport method (85–96% PAH).

According to the maturation measurement intervals, the year was divided into quarters for the assessment of injuries. For each assessment-quarter, injury occurrence was categorised as either no injury or one of the three injury types: (1) *growth-related injuries*, (2) non-contact injuries, or (3) contact injuries. Injury-free quarters were coded as “no injury”. *Growth-related injuries* were defined as “unique injuries not seen in adults, including growth plate fractures, apophysitis, apophyseal avulsion fractures, and greenstick fractures”^19^. Injuries were classified according to the IOC consensus statement^20^ by the same doctor and the lead physio therapist who were both blinded to the players’ maturation status. Injuries were assigned to the nearest assessment. Measurements were carried out exactly every three months. Every injury was counted only in the measurement period in which it occurred. Present injuries during the assessments played no role on the categorisation of the maturation period a player was in. This was only decided by the results of the assessments. Recurrent injuries were not treated separately.

### Statistical analysis

Only players that had measurements for all methods were included in the analysis to make a valid comparison between the methods possible. Descriptive statistics of the players’ characteristics are presented as means and 95% confidence intervals (CIs). Not all of the players had their maturation assessed four times over the period of this one year and therefore occurred with varying frequency within the dataset. Therefore, the player’s ID was controlled as a random effect within the analyses. For the comparison between the three maturation periods (Pre-PHV, PHV, Post-PHV) across injury outcomes, Bayesian multinomial regression analyses with random effects (*brms* package) were performed^21^. Injury occurrence in each player-assessment-quarter served as the dependent variable and had four categories (0 = no injury, 1 = non-contact injury, 2 = growth-related injury, 3 = contact injury). The maturation period according to each method was the independent variable, and individual assessment-quarters (including injury-free quarters) were the unit of analysis. The three injury categories (non-contact, growth-related, contact) were modelled simultaneously in a single Bayesian multinomial model with a four-category outcome, using “no injury” as the reference category. Flat, non-informative priors for all fixed effects (class = b) of the maturation period, weakly informative Student-t priors (3 degrees of freedom, mean 0, scale 2.5) for the intercepts of the three logit equations, and the same Student-t priors for the standard deviations of the random intercepts for player ID were used. These priors correspond to the default weakly informative priors provided by brms for categorical outcomes. Models were fitted using the NUTS sampler with 4 chains and 2000 iterations per chain (1000 warm-up), resulting in 4000 post-warm-up draws. Convergence was assessed via R-hat statistics (all parameters ≈ 1.00) and effective sample sizes (Bulk_ESS and Tail_ESS). For the comparison between the three maturation periods within the three injury categories, the following R coding was used:

dataset$injury_category <- relevel(as.factor(injury_category), ref = “0”)

model[1-9] <- brm(as.factor(injury_category) ∼ [method 1–9] + (1 | ID),

data = dataset,

family = categorical())

summary(model[1-9])

In case multiple injuries per player occurred during one quarter, the first occurring injury was included in the analyses. Multiple injuries across the study period were possible, whereas each assessment-quarter, regardless of whether an injury occurred, was treated as one observation, coded as either “no injury” or one of the three injury types. Peak height velocity (PHV) was defined as the reference maturation period. Posterior estimates and 95% credible intervals (CrI) from the Bayesian multinomial model were exponentiated into odds ratios (OR), representing the central 95% of the posterior distribution for each parameter. For interpretability in a sports medicine context, we focused on ORs and their 95% CrI. An effect was considered noteworthy when the credible interval did not include one. Odds ratios above one indicate higher odds of the respective injury type versus no injury in the comparison period (Pre-PHV or Post-PHV) relative to the PHV period, whereas odds ratios between zero and one indicate lower odds. Statistical analyses were performed using R and Rstudio (version 4.4.2, R Foundation for Statistical Computing).

## Results

Six hundred and nineteen assessment quarters from 205 male academy football players met the inclusion criteria and were analysed. Player counts across the three maturation periods as well as their chronological and biological age (according to the ultrasonic BAUSport device), standing height, mass, and injuries for each method are presented in Table 1.

**Table 1:** Descriptive characteristics of the players’ measurements, divided into Pre-PHV, PHV, and Post-PHV periods with a total of 619 data points for each method.

Method	Maturation period	*N* (obs/subj)	Chronological age [years] Mean (SD)	Biological age [years] Mean (SD)	Standing height [cm] Mean (SD)	Mass [kg] Mean (SD)	Non-contact injuries Total (per 100 measurements)	Growth-related injuries Total (per 100 measurements)	Contact injuries Total (per 100 measurements)
BAUSport 85–96%	Pre-PHV	304/101	10.4 (1.38)	10.8 (1.64)	143 (9.76)	35.2 (6.70)	14 (4.6)	11 (3.6)	32 (10.5)
	PHV	190/77	13.6 (0.94)	14.3 (0.97)	166 (6.55)	54.3 (8.52)	31 (16.3)	18 (9.5)	14 (7.4)
	Post-PHV	125/57	15.3 (0.59)	16.6 (0.73)	179 (5.76)	70.9 (6.70)	40 (32.0)	2 (1.6)	13 (10.4)
Khamis-Roche 88–96% (short)	Pre-PHV	356/122	10.7 (1.53)	11.2 (1.75)	146 (10.8)	36.6 (7.32)	22 (6.2)	16 (4.5)	36 (10.1)
	PHV	145/64	13.9 (0.75)	14.9 (0.99)	170 (6.23)	58.8 (7.41)	24 (16.6)	12 (8.3)	12 (8.3)
	Post-PHV	118/55	15.4 (0.56)	16.5 (0.86)	179 (6.25)	70.4 (7.85)	39 (33.1)	3 (2.5)	11 (9.3)
Khamis-Roche 85–96% (long)	Pre-PHV	289/100	10.3 (1.32)	10.7 (1.60)	143 (9.40)	34.7 (6.44)	14 (4.8)	10 (3.5)	31 (10.7)
	PHV	212/89	13.5 (0.89)	14.3 (1.21)	166 (7.82)	54.4 (9.37)	32 (15.1)	18 (8.5)	17 (8.0)
	Post-PHV	118/55	15.4 (0.56)	16.5 (0.86)	179 (6.25)	70.4 (7.85)	39 (33.1)	3 (2.5)	11 (9.3)
Mirwald APHV ± 12 months (long)	Pre-PHV	333/116	10.6 (1.47)	11.0 (1.68)	144 (10.1)	35.9 (6.84)	18 (5.4)	16 (4.8)	33 (9.9)
	PHV	137/63	13.6 (0.78)	14.4 (0.90)	167 (5.70)	55.2 (7.11)	19 (13.9)	10 (7.3)	11 (8.0)
	Post-PHV	149/66	15.2 (0.61)	16.4 (0.85)	178 (6.08)	69.6 (7.39)	48 (32.2)	5 (3.4)	15 (10.1)
Mirwald APHV ± 6 months (short)	Pre-PHV	371/123	10.8 (1.56)	11.3 (1.77)	146 (10.9)	37.1 (7.65)	20 (5.4)	18 (4.9)	35 (9.4)
	PHV	64/37	13.6 (0.44)	14.5 (0.53)	168 (4.09)	55.4 (4.86)	8 (12.5)	7 (10.9)	7 (10.9)
	Post-PHV	184/75	15.1 (0.71)	16.2 (0.92)	177 (6.44)	68.2 (7.54)	57 (31.0)	6 (3.3)	17 (9.2)
Moore APHV ± 12 months (long)	Pre-PHV	315/109	10.4 (1.39)	10.9 (1.67)	144 (9.99)	35.6 (6.95)	15 (4.8)	12 (3.8)	32 (10.2)
	PHV	154/68	13.5 (0.66)	14.2 (1.00)	166 (6.66)	53.7 (8.23)	22 (14.3)	15 (9.7)	12 (7.8)
	Post-PHV	150/66	15.3 (0.58)	16.4 (0.87)	178 (6.26)	69.4 (7.57)	48 (32.0)	4 (2.7)	15 (10.0)
Moore APHV ± 6 months (short)	Pre-PHV	359/120	10.7 (1.51)	11.2 (1.75)	146 (10.8)	36.8 (7.53)	20 (5.6)	17 (4.7)	35 (9.7)
	PHV	74/38	13.5 (0.36)	14.3 (0.65)	167 (5.11)	54.1 (5.94)	8 (10.8)	8 (10.8)	18 (24.3)
	Post-PHV	186/74	15.1 (0.70)	16.2 (0.92)	177 (6.47)	68.1 (7.63)	57 (30.6)	6 (3.2)	6 (3.2)
Fransen APHV ± 12 months (long)	Pre-PHV	337/128	10.7 (1.65)	11.2 (1.92)	145 (11.7)	36.9 (9.32)	22 (6.5)	18 (5.3)	32 (9.5)
	PHV	148/73	13.5 (0.98)	14.4 (1.08)	167 (6.96)	55.1 (8.20)	19 (12.8)	9 (6.1)	17 (11.5)
	Post-PHV	134/59	15.3 (0.64)	16.4 (0.87)	178 (6.21)	69.3 (7.52)	44 (32.8)	4 (3.0)	10 (7.5)
Fransen APHV ± 6 months (short)	Pre-PHV	381/137	11.0 (1.70)	11.4 (1.97)	147 (12.2)	38.2 (9.71)	26 (6.8)	21 (5.5)	35 (9.2)
	PHV	63/39	13.4 (0.62)	14.4 (0.76)	168 (5.33)	54.9 (6.22)	8 (12.7)	5 (7.9)	7 (11.1)
	Post-PHV	175/74	15.1 (0.76)	16.1 (0.94)	176 (6.54)	67.7 (7.80)	51 (29.1)	5 (2.9)	17 (9.7)

APHV = Age at peak height velocity; obs = observations; subj = subjects.

Overall, there were 85 non-contact injuries, 59 contact injuries, 31 growth-related injuries, and 444 injury-free assessment-quarters recorded. Within the non-contact injuries, muscle (49%) and ligament injuries (31%) occurred most frequently, mainly located at the groin/hip (31%), upper thigh (28%), and ankle/foot (22%). The contact injuries consisted mainly of bone (51%), muscle (20%), and ligament injuries (15%), predominantly located at the ankle/foot (34%), arms/hands (20%), and knee (17%). The growth-related injuries were primarily bone (74%) and tendon injuries (23%), located at the knee (45%), groin/hip (32%), and lower leg (16%). According to age groups, the non-contact injuries were most prominent in the U16, recording 40% (*n* = 34) of all non-contact injuries in this team. Contact injuries occurred most frequently in the U11, accounting for 22% (*n* = 13) of this injury type in that team. Twenty-six per cent (*n* = 8) of the growth-related injuries occurred in the U14 age group, although this injury type was similarly distributed across the U12-U15 teams (U12: 19%/*n* = 6; U13: 23%/*n* = 7; U15: 16%/*n* = 5). The first growth-related injury was reported in the U9 team (*n* = 1; 3% of all growth-related injuries). Five players suffered two growth-related injuries throughout the year, so that the 31 injuries of this type were distributed among 26 players. Average time loss was 22.6 (± 26.4) days due to non-contact injuries, 37.8 (± 43.7) days after growth-related injuries, 21.4 (± 33.2) days caused by contact injuries, and 24.9 (± 32.7) days across all recorded injuries.

**Fig. 1:**
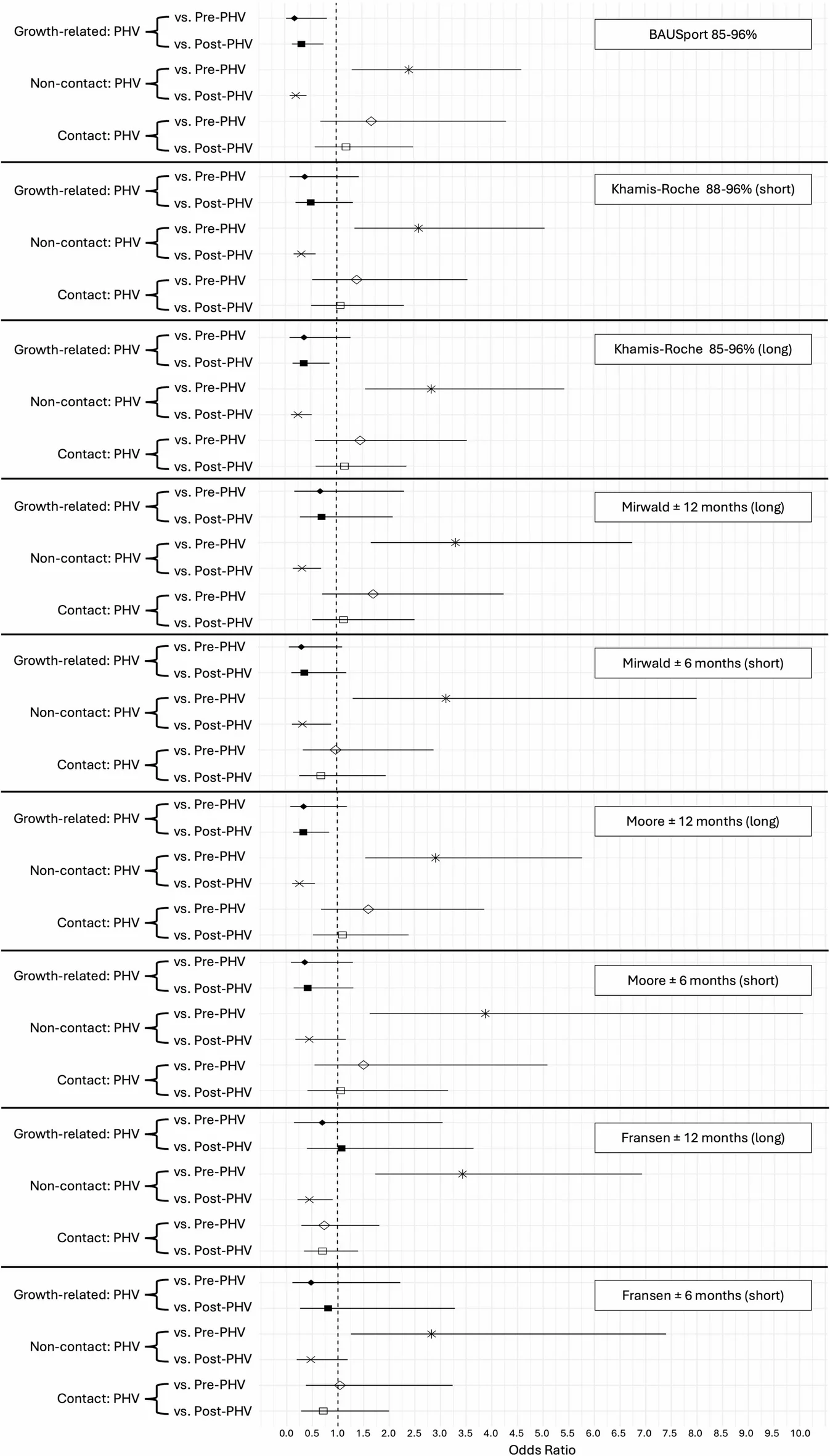
Median and 95% credible interval (CrI) of the odds ratios for the comparison of the PHV period with Pre-PHV/Post-PHV periods, separated for growth-related, non-contact and contact injuries for every method.

The results of the multinomial regression analysis are presented in Fig. 1. In terms of growth-related injuries, the ultrasonic BAUSport, long Khamis-Roche, and long Moore methods were the only methods to indicate significantly lower odds of growth-related injury versus no injury in the Pre-PHV compared to the PHV period. The odds of a growth-related injury versus no injury, as classified by the BAUSport, long Khamis-Roche, and long Moore methods, were 67% (OR = 0.33), 64% (OR = 0.36) and 66% (OR = 0.34) lower, respectively, during the Pre-PHV compared to the PHV period.

The BAUSport method was the only method to indicate significantly lower odds of growth-related injury versus no injury in the Post-PHV compared to the PHV period. According to the BAUSport method, the odds of a growth-related injury were 83% (OR = 0.17) lower during the Post-PHV compared to the PHV period.

All methods, except Moore short and Fransen short, found significantly lower odds of a non-contact injury versus no injury during Pre-PHV than during the PHV period. The odds of a non-contact injury versus no injury in the Pre-PHV compared to the PHV period were 79% lower (OR = 0.21) according to the BAUSport method, 70% lower (OR = 0.30) according to the short Khamis-Roche method, 75% lower (OR = 0.25) according to the long Khamis-Roche method, 67% lower (OR = 0.33) according to the long Mirwald method, 66% lower (OR = 0.34) according to the short Mirwald method, 74% lower (OR = 0.26) according to the long Moore method, and 55% lower (OR = 0.45) according to the long Fransen method.

All methods found significantly higher odds of non-contact injury versus no injury in the Post-PHV period than in the PHV period. The odds of a non-contact injury versus no injury in the Post-PHV compared to the PHV period were 141% (OR = 2.41) higher according to the BAUSport method, 164% (OR = 2.64) higher according to the short Khamis-Roche method, 189% (OR = 2.89) higher according to the long Khamis-Roche method, 229% (OR = 3.29) higher according to the long Mirwald method, 216% (OR = 3.16) higher according to the short Mirwald method, 194% (OR = 2.94) higher according to the long Moore method, and 242% (OR = 3.42) higher according to the long Fransen method.

None of the methods found a significant difference between the three maturation periods in the odds of contact injury versus no injury.

## Discussion

In this cohort, the BAUSport, long Khamis-Roche, and long Moore methods were associated with higher odds of growth-related injury versus no injury during their respective PHV-based Red-Flag period than before this period. Furthermore, the ultrasonic BAUSport method’s identified PHV period was also associated with higher odds of growth-related injury versus no injury than the Post-PHV period. Thus, the BAUSport-derived Red-Flag range showed a distinctive pattern of association with growth-related injuries compared with the other methods, as it was the only approach to indicate lower odds of growth-related injuries versus no injury in both Pre- and Post-PHV periods relative to its PHV period.

The overall injury frequency and distribution recorded in this study can be regarded as representative, as a meta-analysis of 43 studies on youth football players indicates more non-contact than contact injuries as well as a similar distribution of affected physical structures and body regions^22^. The distribution of growth-related injuries was also comparable to previous studies with knee injuries being the most frequent followed by injuries in the groin/hip and lower leg regions^17,23^. Injury severity can further be regarded as representative as other studies show average time losses between 20 and 40 days per injury^16,24^.

None of the PHV assessment methods found any differences between maturation periods in the odds of contact injury versus no injury. This suggests that PHV and possibly associated adolescent awkwardness^2,5,25^ might not have a strong influence on the occurrence of contact injuries. Concerning the non-contact injuries, every method except for Moore short and Fransen short identified significant differences between the three maturation periods, indicating higher odds of non-contact injury versus no injury with ongoing maturation of the players. This indication matches previous studies. For example, Johnson et al.^4^ analysed non-contact injuries in relation to maturation status and found more injuries within the more mature groups referred to PHV. Hall et al.^26^ also found more non-contact injuries in biologically older players and even examined no difference between players post PHV and adults. Other authors indicate those assumptions as well, even though their injury categorisation was slightly different^3,5,17^. It is therefore assumed that non-contact and contact injuries in general are not increased during PHV and cannot be clearly associated with Red-Flag ranges of established PHV assessment methods.

Growth-related injuries, being directly linked to PHV, are the injury type that each method’s Red-Flag range is specifically designed to detect. From the results of this study, only the ultrasonic BAUSport method was associated with higher odds of growth-related injuries versus no injury within its Red-Flag range compared to its Pre- and Post-PHV periods. Two methods (Khamis-Roche long and Moore long) were associated with higher odds of growth-related injury versus no injury in their defined PHV period than prior to this range, but did not show statistically higher odds than during the Post-PHV period. Several studies have attempted to identify periods relative to PHV during which players are most prone to growth-related injuries^17,26^. Studies from Monasterio et al.^17^ and Hall et al.^27^ both found growth-related injuries most frequently occur during the Pre-PHV and PHV periods. Hall et al.^26^ calculated the APHV using the Mirwald method in a different way than analysed in this study (Pre-/Post-PHV defined as APHV ± 12 months or more, and PHV defined as APHV ± 6 months), and Monasterio et al.^17^ applied the percentages of predicted adult height using the Khamis-Roche method with a range of 88–95%, also slightly different than the Khamis-Roche methods analysed in this study. The differences in the analysis methods may have been the decisive factor in the fact that no differences between the Pre-PHV and PHV periods were detected in these studies. The results of Monasterio et al.^17^ show a median percentage of predicted adult height for growth-related injuries of 91.2% with an interquartile range (IQR) of 86.7%-95.2%. These findings are consistent with the present study, suggesting that the occurrence of growth-related injuries is better represented by a broader Red-Flag range. A broader Red-Flag range also appears justified when considering the typical age of onset of common growth-related injuries. Ankle/foot injuries seem to occur earlier than knee injuries and, in turn, earlier than injuries located at and around the pelvis^24,28^.

The present study has several important limitations. Due to the lack of precise data regarding each player’s individual exposure time in training and matches, it was not possible to calculate injury incidence per 1,000 h. However, academy players maintained a representative high training level, and training and match participation were broadly comparable among athletes within the same age groups. Consequently, direct comparability with other studies is limited. This major limitation leaves room for other factors that may influence injury occurrence. For example, older players usually train more and play longer and more intense matches, which may contribute to more injuries. Especially for the non-contact injuries, such factors may possibly contribute as an additional parameter to injury odds. Therefore, the observed associations should be interpreted with caution. Chronological age and maturation status were strongly collinear in this cohort, making it difficult to distinguish their independent effects. Moreover, due to the lack of precise data, the study could not control for prior injury as well as height velocity, which has been shown to influence injury risk^16^. The absence of a gold-standard PHV or skeletal maturity reference additionally precludes firm conclusions about method accuracy. Given the relatively small number of growth-related injuries for the statistical approach applied, the effect estimates may be unstable and should be interpreted with caution and regarded as hypothesis-generating. Furthermore, because the PHV windows analysed were practice- and literature-derived operational definitions of varying width, the results reflect the performance of specific method-window definitions rather than maturation estimation methods independent of window choice.

Additionally, because nine methods were compared and multiple tests were conducted, there is a risk of an accumulation of type I errors, even though the study was exploratory in design. This further emphasises that the findings should be interpreted as exploratory and considered hypothesis-generating. Different reasonable modelling choices within the Bayesian model could have also influenced the results, and the use of self-reported parental height limits the validity of the periods defined by Khamis-Roche methods, as self-reported heights may be inaccurate.

Another limitation of this study is the relatively short data collection period of only one year. Within this timeframe, it was not possible to monitor a player’s growth trajectories from pre- to post-PHV, which would have been desirable to minimise interindividual variability in the analysis. However, extended tracking was not feasible, as plans were already in place to screen players during their PHV, to intervene in case of conspicuous findings, and to implement preventive measures against injuries. This approach precluded the possibility of observing injury occurrence without prior intervention.

The lack of a defined transfer window in youth football leads to many transfers during the season. Consequently, some players entered or left the academy mid-season. This was accounted for in the statistical analysis by including player ID as a random effect.

Within the constraints of this single-academy dataset, the ultrasonic BAUSport method identified a PHV classification window that was consistently associated with growth-related injuries among the tested approaches. This pattern is hypothesis-generating and warrants confirmation in independent samples, especially because BAUSport is a proprietary commercial system and access, cost and implementation may differ substantially between settings. Future studies, ideally longitudinal ones, should consider the influence of factors, such as prior injuries and height velocity, adjust for exposure time, and test against a gold-standard reference in larger external cohorts. Further research is also warranted to evaluate other risk factors for growth-related injuries as well as effective prevention strategies, taking assessments of maturity only as a foundation for further more individual diagnostics.

Looking at the maturation assessment methods, they all include measurements of height and mass. Practitioners should take care to use reliable measuring instruments, perform the measurements at the same day time, likely before training to avoid dehydration, and with the players wearing underwear or only football shorts and shirt. All measurements should be carried out by trained staff.

## Conclusion

Among the tested maturity classification methods, the ultrasonic BAUSport-defined Red-Flag period showed a consistent association with the occurrence of growth-related injuries in this single-academy cohort of male youth academy football players. The small number of growth-related injuries, lack of exposure adjustment, and absence of a gold-standard PHV or skeletal maturity reference preclude firm conclusions about method accuracy. Based on the findings of this study, contact and non-contact injuries do not appear to have higher odds or occurrence close to PHV. Further longitudinal studies with observed growth velocity, exposure data, and external validation are required. In addition, studies investigating diagnostic approaches to assess risk factors beyond PHV, as well as effective prevention strategies to protect players from potentially career-impacting injuries, are warranted.

## Data Availability

Research data are not shared. Due to the participants’ club policy, the data that support the findings of this study cannot be shared and are therefore not publicly available.
